# Novel Advances in Dermatitis Herpetiformis

**DOI:** 10.1155/2012/450109

**Published:** 2012-12-19

**Authors:** Paolo Fabbri, Antonino Salvatore Calabrò, Takashi Hashimoto, Alessio Fasano, Marzia Caproni

**Affiliations:** ^1^Section of Dermatology, Department of Medical and Surgical Critical Care, University of Florence, 50134 Florence, Italy; ^2^Section of Gastroenterology, Department of Clinical Physiopathology, University of Florence, Florence, Italy; ^3^Department of Dermatology, Kurume University School of Medicine and Kurume University Institute of Cutaneous Cell Biology, 67 Asahimachi, Fukuoka Kurume 830-0011, Japan; ^4^Mucosal Biology Research Center and Center for Celiac Research, University of Maryland, School of Medicine, Baltimore, MD 21201, USA

Dermatitis herpetiformis (DH) is an inflammatory cutaneous disease with a chronic-relapsing course that presents with pruritic polymorphic lesions mainly distributed in typical areas such as the extensor aspects of the limbs, the sacral region, and the buttocks. There is growing evidence that DH should be considered the specific phenotypic cutaneous expression of a gluten-sensitive enteropathy indistinguishable from celiac disease (CD). Histologically, DH is characterized by subepidermal blister and accumulation of neutrophils and very few eosinophils at the dermal papillae, while the typical immunopathological finding consists in granular IgA deposits along the basement membrane zone, mainly localized at the papillary tips. Recent studies demonstrated that such IgA are directed against epidermal transglutaminase.

However, in the last years, several papers from the literature reported the presence of atypical findings in patients with DH, suggesting that the features described previously probably need a significant revision and leaving many critical points that should be investigated further in the near future. In the present issue, the recent advances of clinical, pathogenetic, and therapeutic aspects of DH will be addressed.

About the clinical presentation, an increasing number of papers have recently described uncommon features in patients with DH, as reviewed in the present issue by V. C. Bonciolini et al. Accordingly, Ohata et al. in their paper reported 91 Japanese DH cases showing atypical distribution of the lesions and, interestingly, showed that only two of the eight patients (25%) with DH were biopsied in their case series exhibited histopathological signs of CD. However, patients with DH may present the widest spectrum of histological findings of enteropathy, ranging from normal-appearing epithelium to a flat mucosa; moreover, patients with DH significantly increased the frequency of chronic atrophic gastritis than healthy controls, as demonstrated by A. Alakoski et al. in this issue.

From a pathogenetic point of view, the presence of a subepidermal cleft with neutrophilic infiltration at the tips of dermal papillae has been considered the histopathologic hallmark of DH, suggesting a major role for neutrophil granulocytes in the pathogenesis of the skin lesions, as described by D. Bonciani et al. and K. Nakajima in this issue. Besides neutrophils, another cell type has been suggested as a major actor in the pathogenesis of DH, namely, CD4+ T cells. The presence of such cells, with a cytokine expression pattern belonging to the Th2 phenotype, has been documented in recent DH skin lesions as well as in the perilesional skin, suggesting their role in the early phases of DH skin inflammation. Moreover, in this issue, Z. Agnieszka et al. focused the attention on apoptosis, a physiological process that has been shown to play a role in several autoimmune diseases.

Finally, in the present issue, A. Fasano reviewed novel therapeutic approaches for DH. Since DH is considered the specific cutaneous manifestation of CD, besides the symptomatic therapies often used in DH patients to control the skin flares at least in the first phases (i.e., dapsone), gluten-free diet (GFD) is still regarded as the only curative approach to the disease. However, a GFD is very hard to comply with, and different approaches are still under investigations.

In conclusion, some of the more recent advances in DH are presented in this issue. DH is a complex disease, where the skin represents a clue for the identification of a gluten-sensitive enteropathy. The knowledge of the different clinical and immunopathological features of the disease as well as the understanding of its pathogenesis may lead to a better care of our patients.

